# Analysis of the air stream flow parameters generated by the positive pressure ventilator—full scale experiment and CFD simulation

**DOI:** 10.1038/s41598-024-57112-z

**Published:** 2024-03-21

**Authors:** Piotr Kaczmarzyk, Daniel Małozięć, Tomasz Burdzy, Bartosz Ziegler, Piotr Krawiec, Anna Dziechciarz, Łukasz Warguła

**Affiliations:** 1 Science and Research Centre for Fire Protection, National Research Institute, 05-420 Józefów, Poland; 2 Institute of Machine Design, Faculty of Mechanical Engineering, Poznań University of Technology, 60-965 Poznan, Poland; 3 Department of Environmental Engineering, Faculty of Civil Engineering and Resource Management, AGH University, Kraków, Poland; 4 Institute of Thermal Energy, Faculty of Environmental and Energy Engineering, Poznań University of Technology, 60-965 Poznan, Poland

**Keywords:** Energy science and technology, Engineering

## Abstract

Positioning the positive pressure ventilator in front of the door opening affects the effectiveness of the rescue operation carried out during a fire. An important factor determining the effectiveness of the positive pressure ventilator is also the layout of the rooms within the gas exchange path and the obstacles present there. The purpose of this article is to assess the feasibility of using analyses such as large eddy simulation (LES) to verify the efficiency of mobile fans under simulation conditions, without the need for time-consuming experimentation (also for complex room volumes of buildings). The article presents a comparative analysis to assess the degree of convergence of flow parameters obtained during an experiment (in a multi-story building) and computational fluid dynamics (CFD) simulations. For volumetric flow rate, convergence was achieved at levels ranging from 0.4% (for 5 m) to 11.5% (1 m), and for pressure values, the differences achieved ranged from 0.6% (5 m) to 30.1% (4 m). This paper demonstrates that the LES model can be used to perform CFD simulations in the area of assessing the performance of a positive pressure ventilator. The article also describes a test methodology for determining the flow parameters of an air stream, which can be used to perform numerical simulations.

## Introduction

Tactical (mechanical) positive-pressure ventilation is carried out by fire protection units using a mobile fan to remove thermal decomposition products^1–3^ resulting from a fire in construction objects^4,5^. These devices can be used to pump smoke using a number of techniques developed by firefighters: positive pressure ventilation, negative pressure ventilation or positive pressure attack^6^. When considering the existing scientific trend, it should be pointed out that scientists from around the world are conducting numerous experimental studies and numerical analyses related to the assessment of the performance and functionality of mobile fans. In 2022 Kaczmarzyk et al. determined the flow characteristics of a mobile fan for selected positioning parameters of the unit (positioning distance and tilt angle)^7,8^. The performed tests showed that the volumetric flow rate increased with distance, but only up to a distance of 5 m. Similar tests were also performed by Lougheed et al. in 2002, who verified the effect of fan positioning distance on its operating efficiency^9^. As a result of the tests, it was determined that the fan worked most efficiently when it was positioned closest to the door opening^9^. There are also publications in the literature related to CFD (Computational Fluid Dynamics) analyses for assessing the flow parameters of mobile fans. Kerber used the Fire Dynamics Simulator (FDS) program to evaluate the flow characteristics of the airflow generated by the mobile fan. In 2006 in his study Kerber measured the characteristics of the free-flow velocity profile of a fan’s airstream at 17 points, located on the surface of the measurement plane. Tests were performed for 3 fan positioning distances of 1.8 m; 2.4 m and 3.1 m, for an impeller pitch angle of 0 degrees. He also performed a CFD numerical analysis for the same conditions. In his study, he showed a convergence of average velocity values for testing and numerical analysis, at 8.7%—for a distance of 1.8 m; 14.7%—2.4 m; and 3.1% for 3.1 m^10^. Similar tests and numerical analysis were performed by Kaczmarzyk et al.^11^. In this case, the team, through experimental studies and CFD analysis, evaluated the convergence of the airflow shape and velocity profile characteristics generated by the mobile fan. For the numerical analysis, FDS was used. The velocity profile was examined for two positioning distances, i.e. 1.5 and 3.0 m, for nine points located in the central part of the measuring plane (situated at the height of the axis of the fan under test) and 23 selected points distributed over a wider range of the measuring plane. The performed analysis showed the smallest difference in average velocity values of 0.18%, for 9 measurement points over a distance of 3 m. The largest discrepancy in average velocity was recorded at a distance of 1.5 m (for 9 points)—17.94%. With regard to the 23 measurement points, at a distance of 1.5 m the discrepancy was 14.1% and for 3 m it was 7.41%. In 2018, Thielens performed an experiment and numerical analysis (in FDS software) on the assessment of airflow inside a multi-storey construction object^12^. In his work, he carried out several simulations to quantify the airflow velocity profile on the surface of the object’s door and window opening. The distributions of flow velocities over the window surface, as presented in the study, obtained from the numerical analysis, are approximately 30% larger than those recorded during the experiment. In his study, the author also evaluated the magnitude of the mass flow rate that was injected into the tested room volume. A value of 5.9 kg/s was recorded in the simulation, while a value of 3 kg/s was recorded in the actual tests. This analysis shows that the discrepancy may be due to the presence of leaks in the tested building^12^. In describing the CFD issues, it should be noted that the FDS programme has also been used to assess the effect of wind on the performance of tactical mechanical ventilation, using the Positive Pressure Ventilation (PPV) technique^13^. In 2017, Panindre et al. carried out a numerical analysis using Fire Dynamics Simulator on the assessment of the effect of wind (0–10 m/s), on temperature and smoke parameters inside a multi-storey building. The results showed that the effectiveness of mechanical ventilation using a PPV fan, decreases as the wind speed supplied to the room under fire increases. The staircase pressures recorded by the authors were characterised by values in test and simulation, respectively: 25 and 27 Pa (floor 2); 20 and 20 Pa (floor 4), 14 and 15 Pa (floor 6); 12 and 11 Pa (roof door)^13^.

In their study, the authors demonstrated that the positioning of fans in the stairwell and the installation of smoke curtains in window openings can increase the flow and pressure values inside the stairwell, which contributes to reducing the heat input and lowering the temperature in the stairwell^13^.

In 2010, Mahalingam et al. conducted a real-scale experiment using a positive pressure ventilator and carried out a numerical analysis using the CFD programme Smartfire^14^. In his work, he verified the velocity profiles of the incoming airflow on the surface of the inlet opening, which is the door to the ventilated volume, and for the window (outlet) opening. For the designated measurement points, Mahalingam et al. showed a convergence of mean velocities, between the experimental tests and the numerical analysis—47% on the door surface and 16% for the window plane.The aim of this study is to assess the feasibility of using LES-type analyses to verify the efficiency of mobile fans under simulation conditions, without the need for time-consuming experiments. In this paper, a comparative analysis was carried out to assess the convergence of flow parameters (volumetric flow rate and static pressure values) between experimental tests carried out in a multi-storey building and CFD numerical analysis.

There are many tools for assessing the flow parameters generated by fans. These include, for example, OpenFOAM, Smart Fire, Ansys Fluent^15–18^. The Fire Dynamics Simulator program was used for this study due to the fact that it is a commonly used tool by fire prevention units in Europe. The developed ventilator model can be used to assess the effectiveness of tactical mechanical ventilation in newly constructed buildings, where it is necessary to develop rescue plans for people staying there.

## Material and methods

### Real scale test methodology (multi-storey building)

Field-scale tests were carried out using a positive pressure ventilator, a common tool used by fire protection units. The positive pressure fan is a turbo unit, driven by a GX 200 internal combustion engine, maximum power of the engine (at rotational speed 3600 rpm) to 4.1 KW, a displacement capacity 196 cm^3^. For this fan unit, the manufacturer declared a maximum volumetric flow rate of 31,799 m^3^/h^19–21^.

The tests for the assessment of flow parameters were carried out on a 7-storey building from which (by sealing off) four floors were isolated as a gas exchange path. The choice of four floors for the flow tests was dictated by the need to compromise with the staircase-like representation of the test volume and, at the same time, to maintain an appropriate relationship between the measuring range of volumetric flow rate^7^. Test stands—flow resisting curtain (FRC)^22^—were installed in the stairwell window on the south-east wall. The indicated apparatus is a dedicated tool for the evaluation of air jet velocity profiles and volumetric flow rate^8^. The stand was made of aluminium profiles—slides, allowing the measuring module to be transported. A thermo-resistive anemometer is mounted on the module, which moves in a synchronised manner on the surface of the measuring plane, recording air velocity values during this time. The stand is driven by a stepper motor and is controlled using a computer programme that works with a dedicated controller.

Detailed technical aspects of the test stand were also presented by Kaczmarzyk et. al. in other publications^7,8,22^.

The following flow parameters were assessed during the course of this study, i.e.

- velocity of the air stream on the surface of the outlet (measuring plane where 120 measuring points were located), on the surface of which the measuring module moved—a TSI thermo-resistive anemometer with a measuring range of 0.127–50 m/s and an accuracy of 1%);
- Static pressure on each of the 4 floors—this parameter was measured using 4 Setra 265 transducers, which have a measuring range of 0–100 Pa and their accuracy is 0.25% respectively.

**Figure 1:**
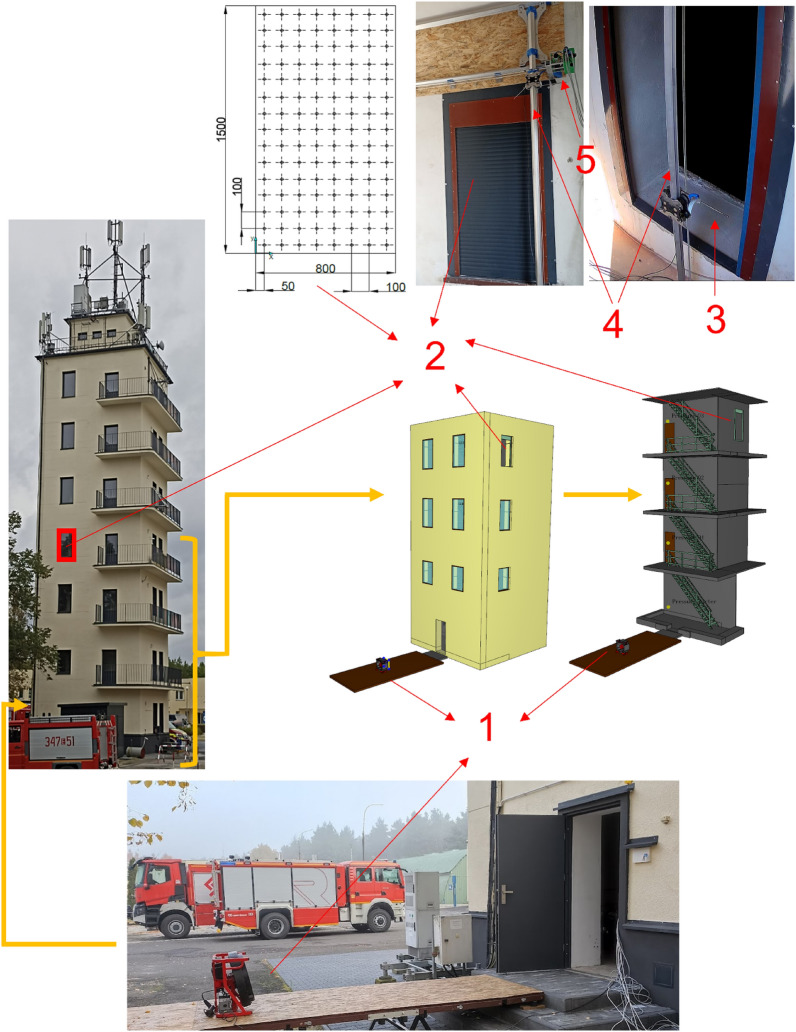
Configuration of the test setup where: 1—positive pressure ventilator, 2—window opening with an area of 1.2 m^2^ (measuring plane with 120 points), 3—measuring probe (TSI thermo anemometer), 4—test stand body and guides for transporting the measuring probe, 5—drive (stepper motor).

A configuration of the test setup and how the equipment and measuring points are arranged is shown in Fig. 1.

The volumetric flow rate was calculated from the product of the velocity and area values, according to the relationship presented by Kaczmarzyk et al.^23^. During the tests, the fan ran at maximum speed. Tests were performed for 6 fan positioning distances, respectively: 1, 3, 4, 5, 6 and 7 m. For each setting distance of the positive pressure ventilator, the angle of inclination was 0°. Environmental conditions were measured during the tests. The test was performed at a temperature of 18 ± 5 °C and a relative humidity of 50 ± 10%. The tests were carried out on days when the wind speed was ≤ 0.2 m/s.

### Methodology for testing air jet velocity profile

**Figure 2:**
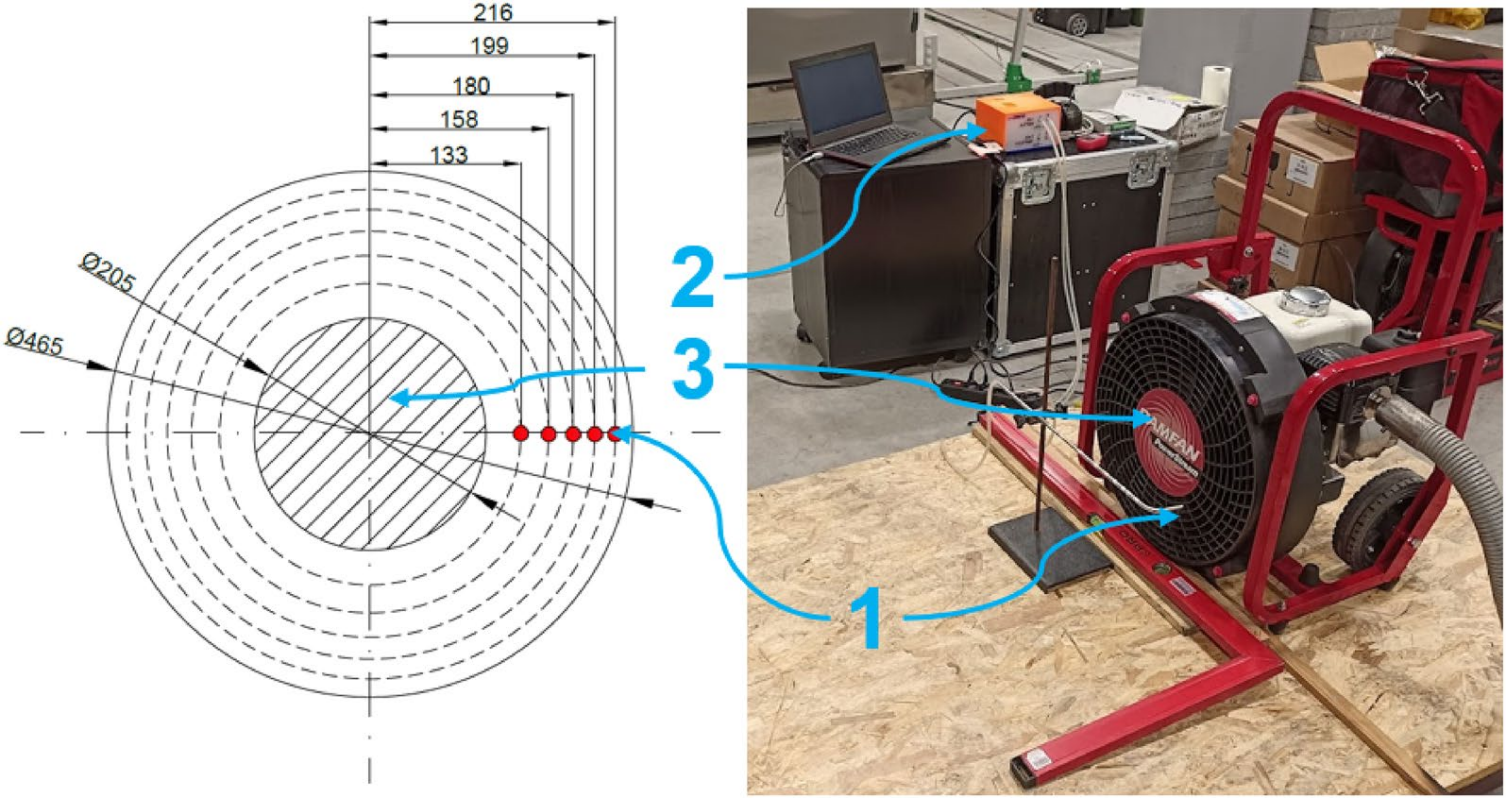
Test configuration for evaluating the flow velocity profile in the direct area of the fan impeller (with an area of 0.17 m^2^), where: 1—measuring point of the flow velocity of the air jet generated by the positive pressure ventilator; 2—setra 265 pressure value transducer; 3—hub on the rotor axis (dead flow area).

As part of this project, a test of the air velocity profile characteristics of a positive pressure ventilator was carried out for the purpose of introducing meaningful flow parameters into the CFD numerical simulation model in Fire Dynamics Simulator. A fan unit that had been subjected to field tests in a multi-storey facility was used for the study. The tests were carried out in the immediate vicinity of the rotor, based on the ISO 5221^24^ standard. Selecting the location of the measuring points on the circular rotor installation plane was based on the modified Log-Chebyshev method^24,25^. The modification of the standard method was related to the isolation of the dead area on the fan unit rotor (positive pressure ventilator hub plugs). No traversal of the flow velocity field was performed on this plane and it was not taken into account for the flow rate calculations. The location of the measurement points and the test configuration of the flow velocity profile are shown in Fig. 2. The following measurement equipment was used to carry out the tests in question:

- prandtl probe made in accordance with the requirements of the ANSI/AMCA 210–16 standard^26^;
- Setra 265 pressure transmitters with a measuring range of 0–1245 Pa and an accuracy of 0.25%;
- Dataq instruments acquisition system (DI-710-UL 0-10 V card);
- supporting tools: protractor, calliper, tape measure and tripod for stabilising the prandtl tube.

The tests were carried out in a symmetrical arrangement (on both sides of the fan impeller) for two probe (prandtl probe) positioning distances in front of the impeller surface, i.e. 50 and 100 mm. During testing, the fan was running at maximum speed. The volumetric flow rate was calculated from the product of the average flow profile values and the fan impeller area^23^. Tests were conducted inside a test hall with a volume of approximately 1500 m^3^, where stable environmental conditions prevailed: temperature 22 ± 1 °C and humidity 50 ± 2%.

### Methodology of numerical CFD analysis

The numerical analysis was performed using FDS (version 6.7.9), an open source CFD software package designed for modelling low-speed turbulent flows with simulation of large LES vortices. The programme was developed by the National Institute of Standards and Technology (NIST)^27^. FDS solves LES equations (spatially and temporarily filtered Navier Stokes equations) to directly solve for large turbulent scales which are responsible for most of the turbulent mass and momentum transport. This approach is particularly suited for buoyancy driven and largely separated (or free) flows. In present study, the flow is mainly driven by a ventilator. As the temperature of the outside air and building walls in the experiment are not identical some input of buoyancy is present. It is however practically impossible to provide the exact building temperature distribution for each analysis, so in the numerical analyses the isothermal flow model is applied. This allows interpreting the CFD results purely as an effect of forcing by a ventilator and building flow resistance.

Subgrid scale momentum transport is achieved with an eddy viscosity model based on the Deardoff model^28^. Details of SGS implementation are given in the FDS technical reference guide^29^.

FDS uses explicit time stepping with automatic timestep control to maintain maximum CFL (Courtant-Friedrischs-Lewy condition) in the domain between 0.8 and 1.0^29^. Poisson equation for pressure is solved by a direct FFT-based solver from CRAYFISHPAK library^29^. Velocity update, and pressure equations are solved repeatedly for each timestep until the maximum velocity error at boundaries drops below a defined tolerance (set to 0.001 m/s).

Validation of the programme to accurately predict smoke flow, including fire conditions, has been performed in full-scale experiments^13,30,31^. Smokview software developed by NIST^32^ and PyroSim version 2022.3.1208 were used to visualise the described model.

To perform the simulation in FDS, a spatial model of the construction object, the external environment and the positive pressure ventilator were created. The spatial model was built on a Cartesian computational grid divided into 2,814,920 computational cells, measuring 0.05 × 0.05 × 0.05 m. The model defines a geometry that corresponds to the actual conditions and sizes of both the 4-storey building and the fan itself. The level of detail of all elements in the model, including the positive pressure ventilator housing, the stairwell and the current obstacles in the corridors, the barriers, the step, the openings in the ceiling, were made to an accuracy corresponding to the resolution of the grid cell, i.e. 5 cm.

**Figure 3:**
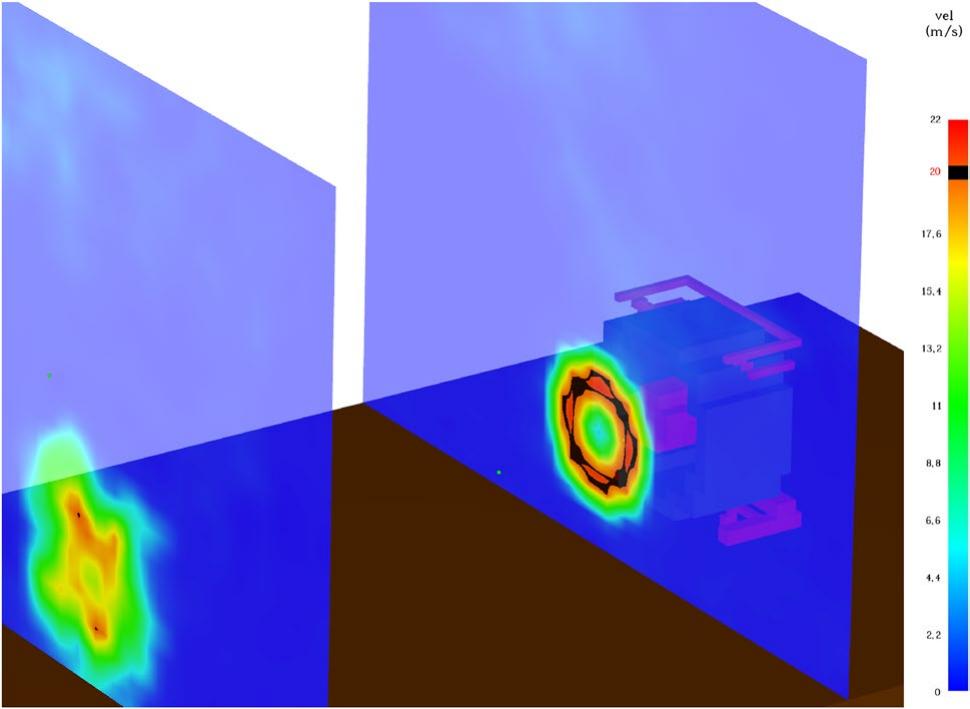
View of the modelled PPV fan based on the ‘velocity patch’ function for the study.

Referring to the modelling of the fan, the flow pattern of the airflow generated by the positive pressure ventilator is described on the basis of the ‘Velocity Patch’ function^33^. In case of the polynomial coefficients representing the velocity values, it is indicated that they were selected for the fan housing Fig. 3 so that a flow rate of 3.5 m^3^/s (Table 3) was obtained when measuring the volumetric efficiency in the CFD analysis (set directly on the impeller surface of the modelled fan). During the CFD analyses, the number of iterations for pressure was increased to a value of 50 and the function that suspends iterations for the default pressure value difference factor in the new time step relative to the value in the previous time step was disabled. These operations were performed in order to increase the accuracy of pressure representation in CFD analyses relative to measured values.

**Figure 4:**
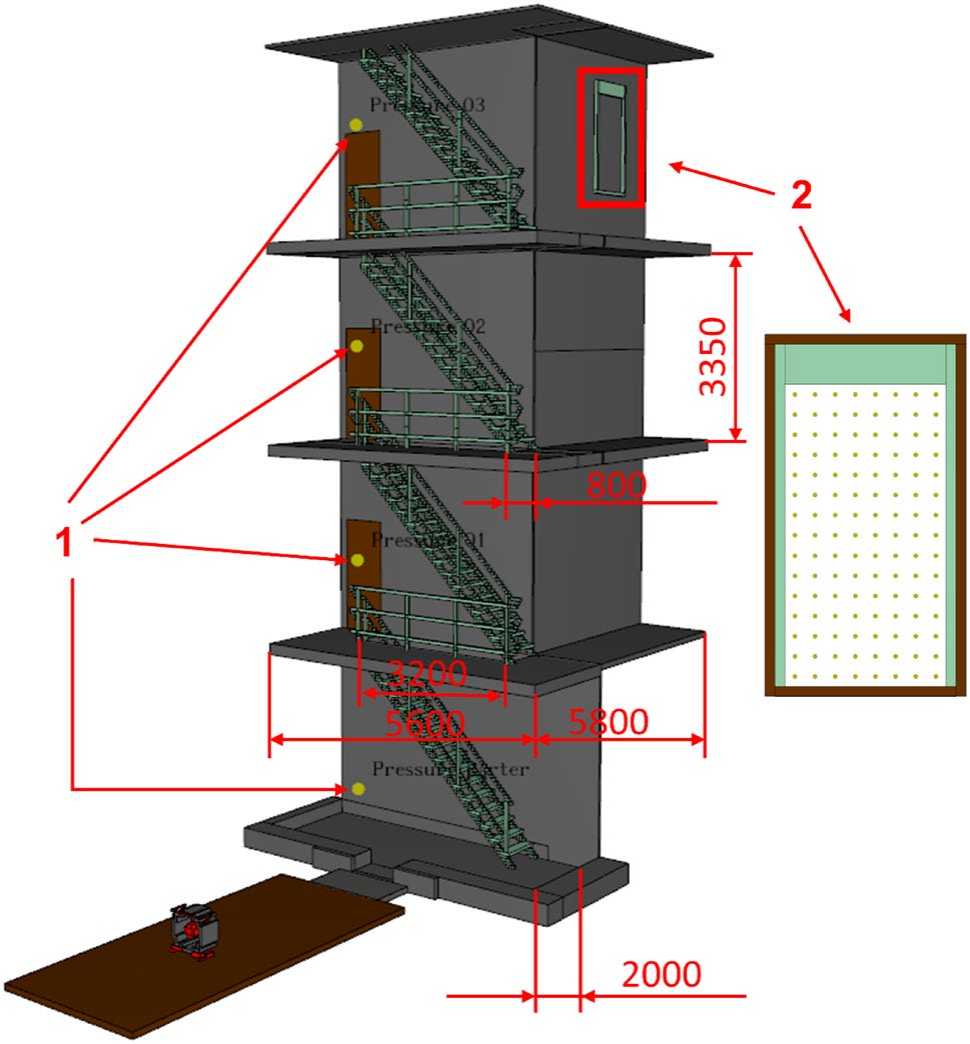
Visualisation of the numerical model built to perform CFD simulations in Fire Dynamics Simulator; where: 1—static pressure value sensor; 2—window opening—measurement plane (with an area of 1.2 m^2^) of air jet velocity values (120 measurement points).

The model is equipped with: reading points:

- velocity of the air stream on the surface of the outlet (window) opening, located on the 4th floor (the same arrangement as in the experimental research) in the number of 120 measurement points [m/s];
- static pressure on each of the 4 floors, measured at the same location as during the actual tests [Pa];

In describing the methodology for using the FDS programme, it should be pointed out that this version allows the creation of a rectangular calculation grid. For this reason, the simulation was performed with a rotor angle of 0° to the ground. Tests were carried out for 6 positioning distances of the positive pressure ventilator (1, 3, 4, 5, 6 and 7 m). The effect of wind was not considered during the simulations due to the fact that the experimental tests were performed in windless conditions. The following environmental conditions were set in the simulation: temperature 20 °C and humidity 50%, pressure 1013 hPa. A visualisation of the model, including its dimensions, is shown in Fig. 4.

Referring to the aspects concerning the acquisition of flow parameters during both test configurations (polygon tests in the construction object and configuration for velocity traversal on the rotor surface) and CFD simulations, in all cases the acquisition (and recording frequency in the case of CFD) was 10 Hz. This value applies to each piece of equipment used during testing, i.e.: pressure transducers, prandtl tube and thermo-resistive anemometer. With the issue of measurement error analysis in mind, it is pointed out that the arithmetic mean was taken as the estimator of the desired value, and the standard deviation of the arithmetic mean was taken as the error of the estimator. Whereas the main test results provided average values of air flow rates from 120 trials (N = 120), for which confidence intervals were determined at a confidence level of 95% (p = 0.05). Significant statistical differences were analysed using Student’s t-test.

## Result and discussion

The results of the tests of the air jet velocity profile on the rotor surface of the positive pressure ventilator are presented in Tables 1 and 2. Meanwhile, the volumetric flow rate values, determined from the flow velocity (from the relationship presented in Kaczmarzyk el al. in 2023^23^), are shown in Table 3. The flow parameters (volumetric flow rate and static pressure values) obtained from the experimental tests and the CFD numerical simulation are shown in Table 4. The presented parameters include the volumetric flow rate and overpressure values inside the ventilated volume, broken down into an average value from all floors and an individual value assigned to each of the 4 levels, for all the positioning setups analysed. Tables 1, 2, 3 and 4 also provide information on the accuracy of the performed measurement.

As a result of the performed work, based on the ISO 5221 standard (measurement of the air velocity profile at the impeller surface), it was observed that the fan (performing the measurement at a distance of 50 mm) obtained the highest velocity of 34.42 m/s, while the lowest measured value was equal to 6.0 m/s. At a distance of 100 mm, velocity values ranged from 6.05 m/s to 31.95 m/s. Referring to the volumetric flow rate values, it is indicated that the fan pumped a flow rate of 12,541 ± 404 m^3^/h (measured at a distance of 50 mm) and 12,482 ± 314 m^3^/h (for 100 mm). Mahalingam et al. in 2010 and Kerber and Walton in 2003 tested 0.75 kW fans (at 2200 rpm) obtaining flow velocities in the similar range of 17.9 m/s and flow rates of 23,900 m^3^/h^14,30^. In 2001, Svensson et al. tested fans with a higher power of 3.73 kW showing an air flow rate of 9,720 m^3^/h^34^. Whereas Weinschenk et al. in 2011 studied the highest-powered fan in the midst of the studies available in the literature, 4.85 kW (at 3500 rpm), which had an airflow rate of 47,232 m^3^/h^35^.

**Figure 5:**
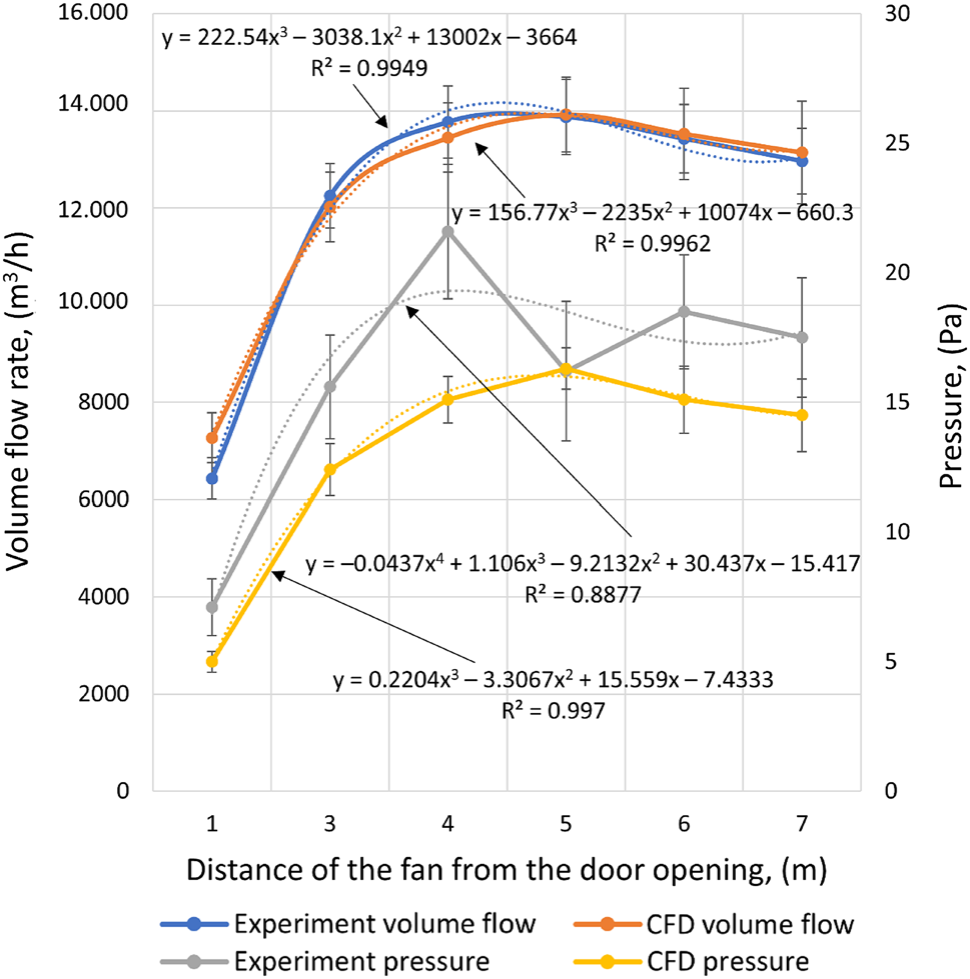
Flow characteristics of the volumetric airflow rate of static pressure obtained from the experiment and CFD simulations.

The comparative characteristics of the flow parameters for the positive pressure ventilator under real and simulated test conditions is shown in Fig. 5. Referring to the obtained values of the flow parameters, it is indicated that the fan generated the highest flow when the fan was positioned at a distance of 5 m. With this configuration setup, it achieved a flow rate of 13,876 ± 775 m^3^/h (at an average pressure of 16.2 Pa) during the experimental tests. On the other hand, during the CFD simulation for 5 m, the fan generated a flow rate of 13,926 ± 773 m^3^/h (and an average pressure of 16.3 Pa). Describing the least favourable setting of the fan, the authors’ team indicates that it worked least effectively when set at a distance of 1 m. Another observation for the 1 m distance case, is that the relative difference between experiment and CFD is highest in this case. This might be the result of the small effectiveness of the ground floor pressure generation by a PPV positioned this close, as a result the unaccounted buoyancy forces from temperature differences play the highest role in this case.

**Figure 6:**
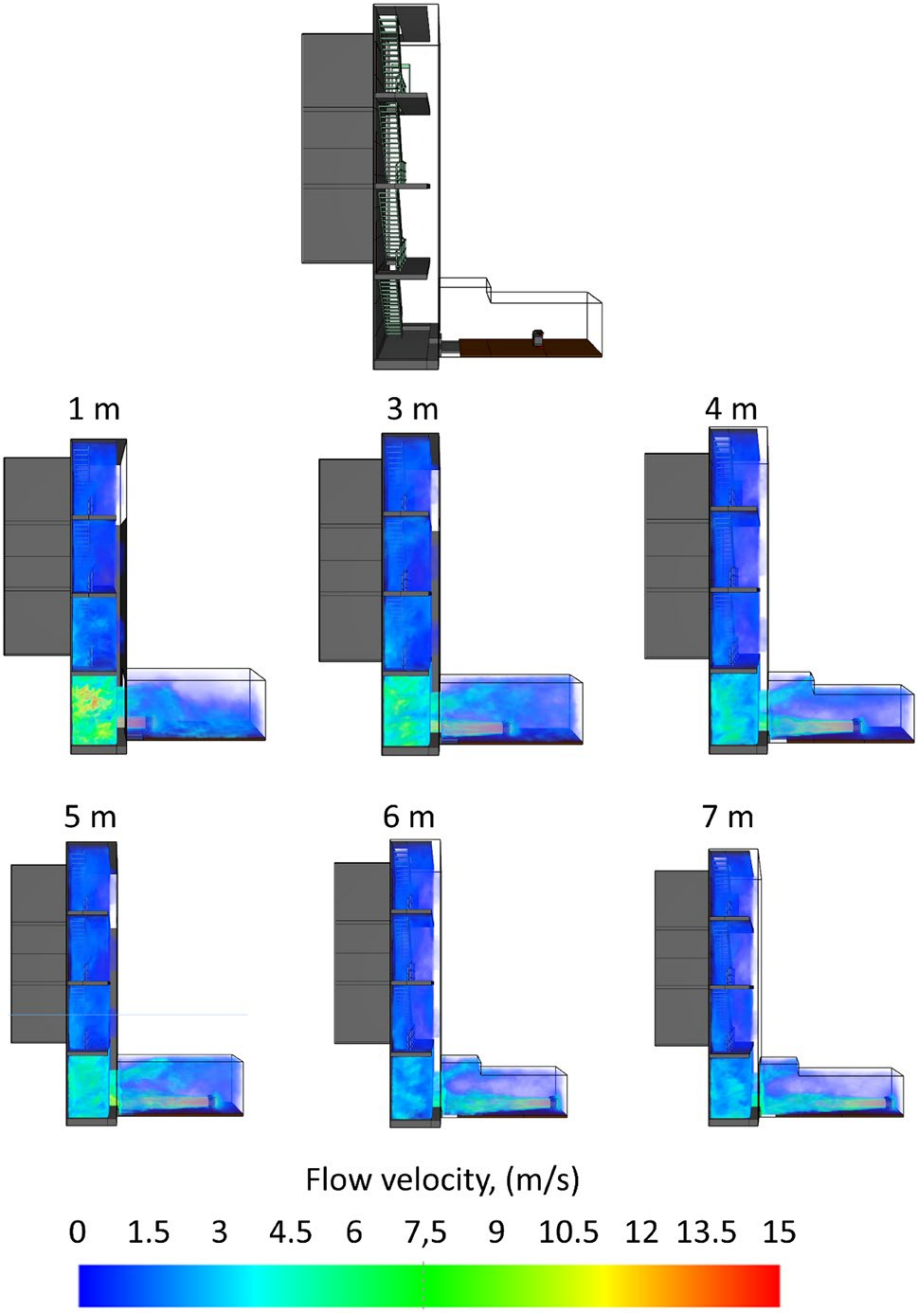
Visualisation of CFD simulation (before and after stabilisation)—distribution of air velocity profiles generated by the PPV fan for positioning distances from 1 to 7 m.

The above relationship applies to experimental studies and simulations. At the distance in question, the positive pressure ventilator achieved an intensity of 6.432 ± 427 m^3^/h and an average pressure of 7.1 Pa during the test, and 7.270 ± 514 m^3^/h and 5.0 Pa during the simulation, respectively. Figure 6 shows a visualisation of a CFD simulation, of airflow through the volume of a building, for positioning distances of 1, 3, 4, 5, 6 and 7 m. As a result of the comparative analysis of the model (between the experimental studies and the CFD analysis), for the volumetric intensity, discrepancies were noted ranging from: 0.4 per cent (for 5 m) to 11.5 per cent (over a distance of 1 m). Analysing the pressure values, it is indicated that in this case there were higher discrepancy values, which ranged from 0.6% (for 5 m) to 30.1% (for 4 m). A study by Ezekoye et al. in 2007 of fans ranging from 2.98 kW to 4.85 kW (with rated flow rates ranging from 25,092 to 48,600 m^3^/h) shows that the 4.85 kW fans exhibit the highest flow rates above a distance of 2.5 m and maintain them up to a distance of at least 3.5 m (the authors did not investigate further)^36^. In a study by Ingason and Fallberg in 2002, for a 4.1 kW fan, the most favourable placement distance is above 3 m, no studies were conducted at further distances, while no downward trend was observed^37^.

**Figure 7:**
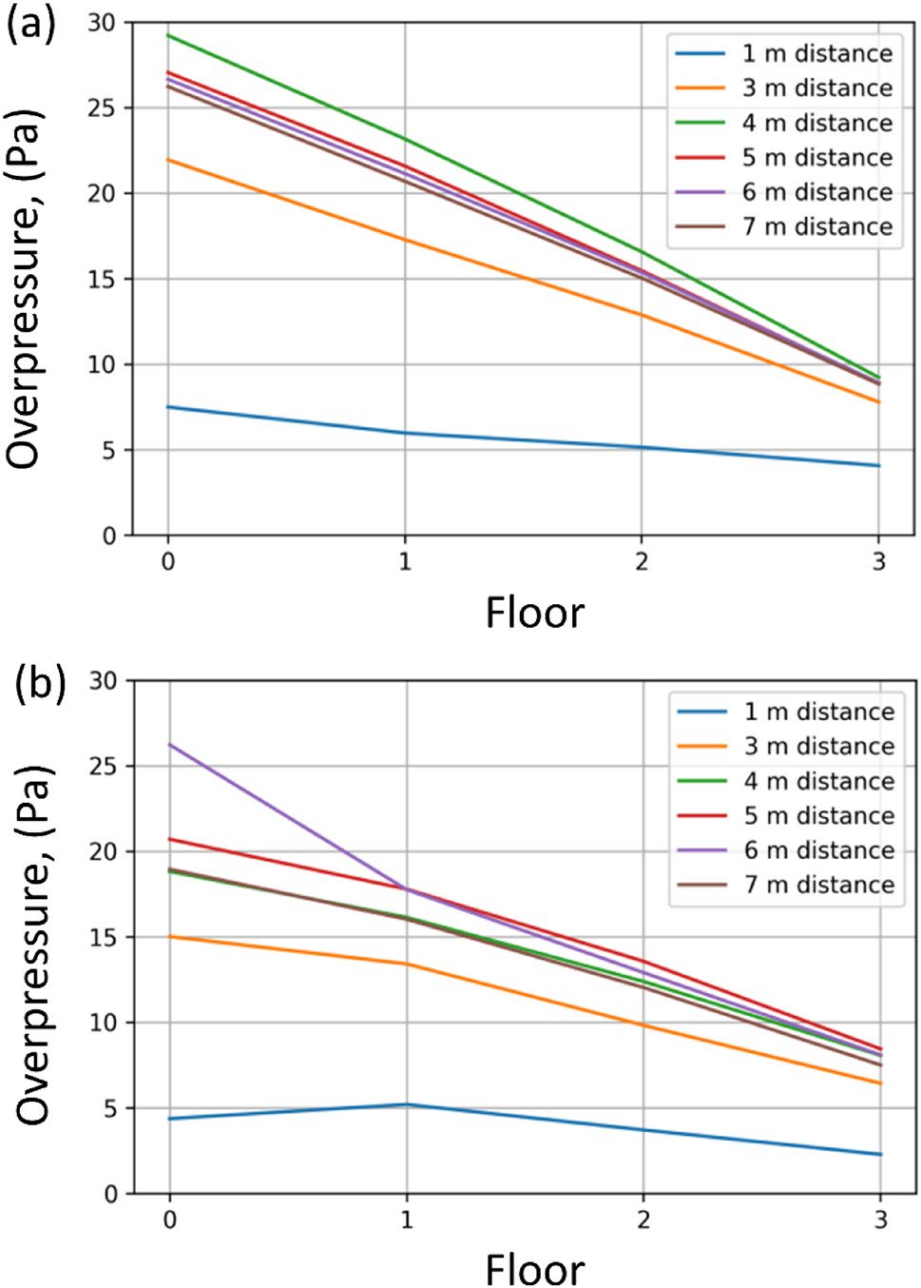
Overpressure fluctuations on successive floors in experimental tests and CFD simulations, (**a**) experimental pressure distribution, (**b**) CFD pressure distribution.

The graph shown in Fig. 7 illustrates the change in pressure values at successive floors in the experimental tests and CFD analysis. In both cases (experiment and CFD), pressure drops on successive floors are similar to each other suggesting a similarity of the flow field on each floor and an almost linear dependence of the building pressure loss coefficient on the number of floors through which air is pumped. A similar relationship is shown by Paninder et al.^13,38^ and Li et al.^39^.

**Figure 8:**
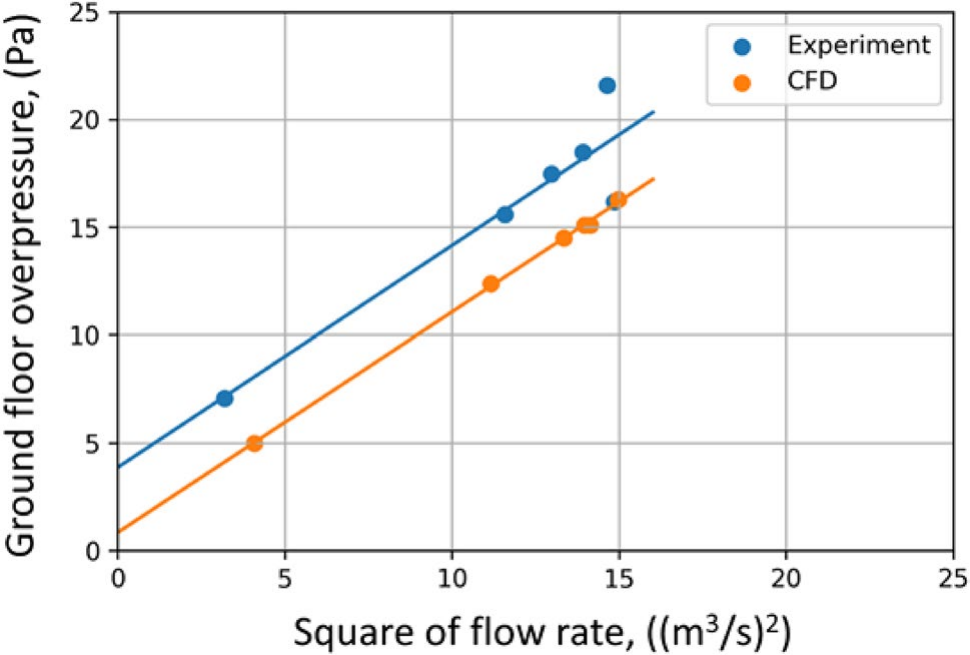
Dependence of the change in overpressure on the first floor as a function of the square of the volume flow rate.

For pressure losses of a local nature, i.e. those associated with the conversion of pressure into dynamic pressure (flow velocity) in constrictions such as doorways or openings between floors, the value of the pressure loss is proportional to the square of the flow velocity and thus to the square of the flow rate. The graph in Fig. 8 shows the relationship of the change in overpressure on the ground floor of the building (and thus also flow losses) as a function of the square of the volumetric flow rate. Beyond the points, the graph shows linear regressions of both characteristics. Their slope coefficients are 1.029 Pa*s^2^*m^−6^ for the experiment and 1.023 Pa*s^2^*m^−6^ for the numerical analysis, respectively. Such close values suggest that the methodology studied for analysing flow through a building allows relatively accurate estimation of flow losses in buildings. The difference in the free expressions of the two regressions lines is 3.02 Pa. It is estimated that this difference is mostly an effect of buoyancy forces not included in CFD. For the height of the building, that pressure difference corresponds to temperature differences of 5–6 K. In practical applications, assessing this buoyant pressure component is impractical as it is a result of air temperature distribution along the flow, however as shown here the isothermal analysis is a useful tool in assessment of the building flow resistance and optimal positioning of the fan what is of the highest importance for the end user.

## Conclusions

Selecting the correct positioning of the fan in front of the inlet opening (facility door) has a significant impact on the volumetric flow rates, which can determine the effectiveness of fire gas migration during rescue operations. A means of assessing the efficiency of a fan to select the appropriate placement distance can be done using CFD tools with the LES model. For the selected turbo fan, a comparative analysis was carried out to assess the degree of convergence between the flow parameters obtained from the experiment (in a multi-storey building) and the CFD simulation in the FDS programme. The performed analysis confirmed the convergence of the flow parameters between the experiment and the CFD analysis with a particular focus on the relationship of pressure loss and flow rate through the building. For volumetric flow rate, convergence was achieved between 0.4% (for 5 m) and 11.5% (1 m), while for pressure values it was achieved between 0.6% (5 m) and 30.1% (4 m). This publication proposes a means of assessing air velocity profile testing for mobile fans. This method can be used to determine the airflow parameter for performing CFD simulations in the LES model. The performed study confirmed the feasibility of using LES (Large Eddy Simulation) type analyses to verify the efficiency of mobile fans under simulation conditions, without the need for time-consuming experimentation (also for complex room volumes of buildings).

## Data Availability

The datasets generated and/or analysed during the current study are available in the Analysis of the air stream flow parameters generated by the positive pressure ventilator—full scale experiment and CFD simulation repository, https://drive.google.com/drive/folders/1YTWZ7FIKN9YGf2AFRimDaK9xL-hw-OAQ?usp=sharing.

## Appendix

See Tables 1, 2, 3 and 4.

**Table 1:** Flow velocity of the air jet generated by the positive pressure ventilator, measured at the rotor surface at a distance of 50 mm.

Sampling location on the impeller surface	Air flow velocity at the indicated sampling points (distance 50 mm) [m/s]									
	± 216		± 199		± 180		± 158		± 133	
	AVG	SD	AVG	SD	AVG	SD	AVG	SD	AVG	SD
Left side	6.00	1.48	22.81	0.71	28.18	0.36	27.84	0.98	34.42	0.70
Right side	11.43	0.95	28.61	1.36	33.10	0.44	29.91	0.39	26.52	0.65

AVG, arithmetic mean of the flow rate; SD, standard deviation taken as the measurement error.

**Table 2:** Flow velocity of the air jet generated by the positive pressure ventilator, measured at the rotor surface over a distance of 100 mm.

Sampling location on the impeller surface	Air flow velocity at the indicated sampling points (distance 100 mm) [m/s]									
	± 216		± 199		± 180		± 158		± 133	
	AVG	SD	AVG	SD	AVG	SD	AVG	SD	AVG	SD
Left side	6.05	1.19	18.48	0.85	28.91	0.50	31.06	0.36	30.61	0.51
Right side	13.89	0.74	23.41	0.54	31.95	0.42	31.74	0.39	31.56	0.73

AVG, arithmetic mean of the flow rate; SD, standard deviation taken as the measurement error.

**Table 3:** Value of the volumetric flow rate generated by the positive pressure ventilator, measured at the rotor surface at distances of 50 and 100 mm.

Measurement distance from the rotor Surface [mm]	Flow rate obtained by positive pressure ventilator [m^3^/h]	
	AVG	SD
50	50	12,541
100	100	12,482

AVG, arithmetic mean of flow rate; SD, standard deviation taken as measurement error.

**Table 4:** Volumetric air flow rate and static pressure values for full-scale experiment and CFD simulation (with variable PPV fan positioning parameters).

PPV setting distance [m]	Case	Volumetric flow rate (m^3^/h)		Static pressure—averaged for 4 storeys (Pa)		Pressure value on the floors of the analysed storeys (Pa)							
						1		2		3		4	
		AVG	SD	AVG	SD	AVG	SD	AVG	SD	AVG	SD	AVG	SD
1	Experiment	6431.9	427.1	7.1	1.1	9.0	1.5	7.7	1.1	6.5	0.9	5.1	0.7
	CFD	7269.6	–	5.0	–	8.5	–	5.3	–	3.8	–	2.3	–
	Difference [%]	11.5%	29.6	5.6	31.2	41.5	45.1						
3	Experiment	12,252.8	662.2	15.6	2.0	22.8	2.7	17.9	2.2	13.4	1.8	8.2	1.2
	CFD	12,024.1	–	12.4	–	19.4	–	13.8	–	9.9	–	6.4	–
	Difference [%]	1.9%	20.5	11.6	22.9	24.2	21.5						
4	Experiment	13,774.1	743.6	21.6	2.6	31.8	3.4	25.4	2.8	18.5	2.4	10.9	1.7
	CFD	13,449.1	–	15.1	–	23.0	–	16.9	–	12.4	–	8.0	–
	Difference [%]	2.4	30.1	34.0	33.5	33.0	26.6						
5	Experiment	13,875.5	775.7	16.2	2.7	24.0	3.7	19.5	3.1	13.7	2.4	7.8	1.5
	CFD	13,925.7	–	16.3	–	24.6	–	18.4	–	13.7	–	8.5	–
	Difference [%]	0.4	0.6	2.4	5.6	0	8.2						
6	Experiment	13,430.8	703.9	18.5	2.2	27.4	3.1	21.8	2.5	15.8	1.9	9.2	1.3
	CFD	13,530.0	–	15.1	–	21.7	–	15.9	–	11.2	–	11.6	–
	Difference [%]	0.7	18.4	20.9	27.1	29.1	20.7						
7	Experiment	12,969.1	673.5	17.5	2.3	26.1	3.2	20.4	2.6	14.8	1.9	8.6	1.2
	CFD	13,145.3	–	14.5	–	21.7	–	16.4	–	12.1	–	7.6	–
	Difference [%]	1.4	17.1	16.9	19.6	18.2	11.6						

AVG, arithmetic mean of flow rate; SD, standard deviation taken as measurement error.
